# Second‐Life Lithium‐Ion Batteries for Circular Energy Systems: A Techno‐Economic and Environmental Pathway to Affordable Grid‐Connected Renewable Storage

**DOI:** 10.1002/gch2.70150

**Published:** 2026-08-21

**Authors:** Abdelhak Lekbir, Bessam Deboucha, Abdullahi Mohamed Samatar, Saad Mekhilef, Kok Soon Tey, Mehdi Seyedmahmoudian, Khalid Alqunun

**Affiliations:** ^1^ Department of Computer System & Technology Faculty of Computer Science and Information Technology Universiti Malaya Kuala Lumpur Malaysia; ^2^ Power Electronics and Renewable Energy Research Laboratory (PEARL) Department of Electrical Engineering Universiti Malaya Kuala Lumpur Malaysia; ^3^ Department of Electrical Engineering Faculty of Engineering Hormuud University Mogadishu Somalia; ^4^ School of Engineering Swinburne University of Technology Hawthorn Victoria Australia; ^5^ Department of Electrical Engineering College of Engineering University of Ha'il Ha'il Saudi Arabia

**Keywords:** circular energy economy, grid‐connected storage, hybrid renewable energy system, levelized cost of storage, second‐life lithium‐ion batteries, techno‐economic analysis

## Abstract

The rapid electrification of transportation is generating a growing stream of retired electric vehicle batteries, creating challenges in battery waste management and the provision of affordable energy storage for renewable energy integration. Repurposing these batteries for stationary applications offers a circular‐economy solution that addresses both issues. This study evaluates the techno‐economic and environmental viability of integrating containerized second‐life lithium‐ion batteries (SLBs) into a grid‐connected hybrid renewable energy system (HRES) for a residential community in Kuala Lumpur, Malaysia. Using HOMER Pro, four configurations incorporating either new or second‐life batteries were compared. The results show that the HRES‐4 configuration reduces grid dependency by more than 54%, transforms the system into a net energy exporter (>820 MWh/year), and achieves the best economic performance, with a net present cost of $239.6K and a levelized cost of storage of $0.072/kWh. Despite higher degradation rates, SLBs deliver energy throughput and cycling performance comparable to new batteries, exceeding 207 MWh/year and 230 equivalent full cycles annually. SLB integration also avoids approximately 1161 tons of CO_2_/year, with a carbon payback of 7.13 years. These findings show that containerized SLBs offer a cost‐effective and scalable pathway for circular energy systems, with relevance to circular‐economy policy and emerging power grids.

## Graphical abstract

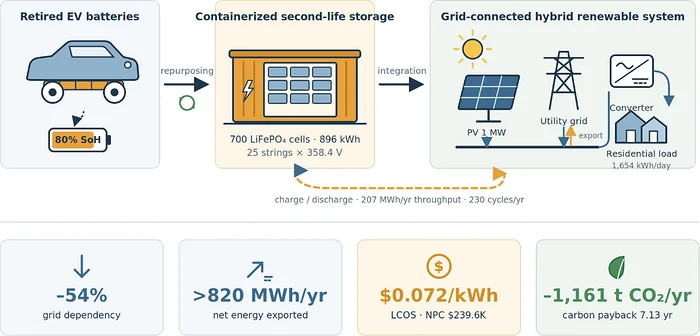

## 1 Introduction

The rapid growth of global energy demand, driven by population expansion, urbanization, and industrial development, continues to intensify pressure on conventional energy systems and accelerate environmental degradation [1, 2, 3]. Renewable energy (RE) technologies have emerged as a central pathway for decarbonizing the power sector and achieving long‐term sustainability goals [4, 5, 6]. The deployment of RE‐based power systems has become particularly important for off‐grid and remote urban communities, where reliable access to electricity remains a critical challenge and dependence on fossil‐fuel‐based generation contributes to high operational costs and environmental impacts [7, 8, 9]. In parallel with the expansion of RE systems, the rapid electrification of the transportation sector has significantly increased the number of electric vehicles (EVs), leading to a growing volume of end‐of‐life lithium‐ion batteries (LIBs) [10, 11]. Typically, EV batteries are retired after 8–10 years of operation when their capacity declines to approximately 70%–80% of the original value [12, 13]. Despite this degradation, these batteries retain considerable residual capacity, making them suitable for secondary applications in stationary energy storage systems. This has led to the emergence of second‐life batteries (SLBs), which support circular economy principles by extending battery lifecycles and reducing environmental burdens associated with premature disposal [14]. The rapid expansion of the electric vehicle (EV) market further reinforces the growing importance of battery lifecycle management. The number of EV registrations surpassed 6.6 million globally in 2021 alone, accounting for approximately 9% of total car sales. With continued policy incentives and infrastructure development, the EV market is projected to reach a valuation of approximately $866 billion by 2028, growing at a compound annual growth rate of 47.3% [15]. This accelerated adoption is expected to generate a substantial volume of end‐of‐life lithium‐ion batteries. It is estimated that between 2025 and 2030, the annual volume of retired automotive batteries will reach 100–200 GWh, a scale comparable to the energy storage capacity added by newly deployed EVs each year [16]. This growing volume of retired batteries creates both an environmental challenge and a significant opportunity for second‐life energy storage applications.

The integration of SLBs into hybrid renewable energy systems (HRES) offers a promising way to enhance system flexibility while reducing capital investment. Previous studies have demonstrated that SLBs can effectively support applications such as peak shaving, load leveling, and RE smoothing under moderate operating conditions [12, 13]. Economically, SLBs offer cost advantages over new batteries, with reported reductions in the levelized cost of energy (LCOE) ranging from 12% to 41% depending on the application scenario [13]. Furthermore, real‐world implementations have shown improved net present value (NPV) and reduced operational costs, highlighting the economic potential of battery repurposing strategies [17]. Environmentally, SLBs help reduce greenhouse gas (GHG) emissions and delay resource‐intensive recycling processes, thereby supporting sustainable energy transitions [18]. However, despite these advantages, the large‐scale deployment of SLBs remains constrained by several technical, economic, and operational challenges. From a technical perspective, second‐life batteries exhibit heterogeneous characteristics, including variations in state of health (SoH), degradation rates, and operational history [19]. These inconsistencies complicate system integration, particularly in series–parallel configurations, and require advanced battery management systems (BMS) to ensure safe and efficient operation [20]. In addition, issues related to thermal management, voltage imbalance, and reliability become more pronounced in densely packed battery systems operating under dynamic load conditions [21].

From an economic standpoint, while SLBs provide lower initial costs, their long‐term viability remains uncertain owing to accelerated degradation under high‐power or high‐cycling applications [22]. In some cases, these factors may offset the initial cost advantages, resulting in a higher levelized cost of storage (LCOS) compared to new battery systems [23, 24]. Furthermore, the integration of SLBs into containerized energy storage systems introduces additional costs associated with power conversion systems, thermal regulation, safety mechanisms, and grid interconnection requirements [24, 25]. Beyond technical and economic considerations, the environmental implications of SLBs require comprehensive evaluation [26]. Life‐cycle assessment studies indicate that SLBs can reduce global warming potential (GWP) by 7%–77% compared to new battery production, depending on the operational lifespan and system configuration [14, 17, 27]. However, these benefits are highly dependent on the second‐life duration, refurbishment processes, and system efficiency. It is important to note that SLBs delay rather than eliminate the need for recycling, emphasizing the importance of integrated lifecycle management strategies [28].

To address these challenges, containerized battery energy storage systems (BESS) have emerged as a practical solution for large‐scale SLB deployment [29]. Containerization enables modular design, improved safety, ease of transportation, and rapid installation, making it suitable for grid‐connected applications [30]. Additionally, standardized containerized systems facilitate compliance with grid interconnection standards and enhance operational reliability. Nevertheless, optimizing the performance of containerized SLB systems requires comprehensive evaluation frameworks that simultaneously consider techno‐economic, operational, and environmental factors [31, 32].

Despite the growing body of literature, several critical research gaps remain. First, most studies focus on either technical performance or economic feasibility, with limited integration of environmental assessment. Second, and more fundamentally, existing HOMER‐based assessments of second‐life storage rarely include a storage‐free photovoltaic (PV) reference case; therefore, the reported benefits cannot be attributed to the PV array and the battery itself. Third, there is a lack of standardized frameworks for evaluating SLB performance within containerized systems under realistic grid‐connected conditions. Finally, limited attention has been paid to the role of SLBs in enabling circular energy systems through delayed recycling and extended‐lifecycle utilization. In this context, this study presents a comprehensive techno‐economic and environmental assessment of integrating containerized second‐life lithium‐ion batteries into a grid‐connected HRES. A detailed modeling framework was developed in HOMER Pro to evaluate multiple system configurations that incorporate both new and second‐life battery technologies. This study aimed to quantify the system performance, economic feasibility, and environmental benefits under realistic operating conditions. The main contributions of this study are summarized as follows:

- Introduction of a storage‐free grid‐PV reference case that isolates the contribution of the second‐life battery from that of the PV array and electricity export, and identification of the mechanism by which storage adds value, namely, the removal of the surplus‐absorption limit that caps the economically deployable PV capacity;
- Demonstration that the economic advantage of storage in this configuration is contingent on the second‐life acquisition cost rather than on storage per se, since the new‐battery configuration returns a 25‐year cumulative cost essentially level with the storage‐free reference;
- Identification of a metric‐level caution for net‐exporting systems, namely that the levelized cost of electricity becomes a small residual between the annualized cost and export revenue and is therefore numerically ill‐conditioned, with annual worth proposed as the more robust indicator.
- Deployment of the analysis within a containerized architecture sized for a specific Malaysian residential community, together with an explicit statement of the boundary, assumptions, and limitations of the environmental assessment; and a direct comparison against recent representative HOMER‐based studies.

## 2 Methodology

This study investigates the impact of integrating a storage container that uses second‐life batteries from EVs on grid system reliability. In this section, the methodology and procedures used to evaluate the techno‐economic performance, environmental impact, and overall benefits of the proposed system are presented.

### 2.1 System Description and Architecture

**FIGURE 1:**
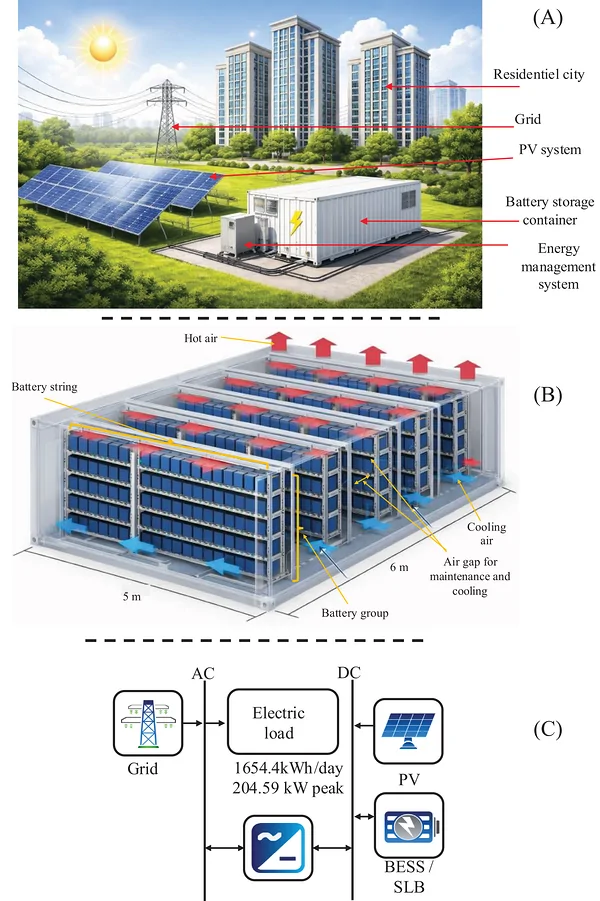
System configuration of the proposed hybrid microgrid: (A) Schematic representation of the microgrid architecture; (B) configuration implemented in HOMER Pro.

The proposed storage system was integrated into an HRES for a residential city located in Kuala Lumpur, Malaysia. The operating weather conditions, including solar radiation and ambient temperature, were obtained from the NASA database using the HOMER Pro software platform. The results indicate that solar radiation ranges from 4.17 to 5.42 kWh/m^2^/day, with a clearness index ranging from 43.7% to 52.9%. The ambient temperature ranges between 24.55°C and 26.05°C. Before evaluating the performance of the proposed HRES, multiple system configurations were analyzed to assess the techno‐economic and environmental impacts of employing a containerized energy storage system based on second‐life batteries. The overall system configuration is illustrated in Figure 1A. In this context, the proposed energy storage system was integrated within a containerized structure to enhance operational safety, improve system mobility, and facilitate maintenance.

The container dimensions were optimized to 6 × 5 × 2 m, enabling the integration of 700 lithium iron phosphate (LiFePO_4_) batteries. This battery technology, widely used in electric vehicles, offers high safety and is compatible with conventional lead‐acid charging systems. The container layout was carefully designed to accommodate battery modules, maintenance space, thermal management (cooling channels), and battery racks for each string. Furthermore, the optimized battery configuration consisted of 25 strings, arranged into five groups, each comprising five vertically stacked strings. Each string included 28 batteries rated at 12.8 V, resulting in a total string voltage of 358.4 V, which is compatible with the standard grid voltage level in Malaysia, as depicted in Figure 1B. The capacities of the PV system and the power converter were optimized using the HOMER software. This tool enables optimal system sizing based on load demand requirements and system constraints. The HOMER‐based optimization relies on an equivalent system representation, as depicted in Figure 1C.

Figure 1C shows the schematic of the HOMER software model for the proposed HRES. The scaled load demand was approximately 1654.4 kWh/day, with a peak load of 204.59 kW, which is typically supplied by the grid in a conventional system configuration. The proposed HRES includes a PV system to enhance the overall system performance and reduce dependence on the grid. In this context, a monocrystalline PV system with a rated capacity of 310 W per module and a conversion efficiency of 19% was selected for integration into the HRES. The hourly electrical energy output of the PV array can be estimated as follows [33, 34]:

(1) $$P_{PV}=Y_{PV}\;\times f_{PV}\left(\frac{G_{T}}{G_{T,STC}}\right)\left[1+\alpha_{P}\left(T_{C}-T_{C,STC}\right)\right]$$

where *Y_PV_*, *f_PV_*, α_*P*_, *G_T_*, *T_C_*, and *STR*denote the PV output power, the derating factor of the PV system, the power temperature coefficient, the incident solar irradiance, the PV cell operating temperature, and the standard test reference conditions, respectively.

The proposed HRES also incorporates a power converter to enhance the system's overall reliability and ensure proper interconnection between the DC and AC buses. The converter consists of two main components: an inverter and a rectifier; therefore, its efficiency depends on the operational performance of these two subsystems. The power rating of the system converter can be optimized as follows [33]:

(2) $$P_{con}=\frac{P_{peak}}{\eta_{con}}\;$$

where *P*_con_, η_con_, and *P*_peak_represent the converter power, converter efficiency, and peak load at the point of consumption, respectively.

The simulation of the proposed system considers different operating scenarios in which the performance of the storage system is analyzed using both new battery banks and second‐life battery banks (used). This was done to demonstrate the feasibility of extending the life cycle of batteries removed from electric vehicles and replaced with new ones. The following subsection provides further details on the energy storage system under consideration. Meanwhile, the simulation methodology introduces four configurations that are compared with two reference cases, as illustrated in Table 1. The first reference is the actual grid‐only system, and the second is a grid‐PV (G‐PV) system without any battery storage. The G‐PV case is deliberately included so that the contribution of the second‐life battery bank can be isolated from the contribution of the PV capacity and the associated electricity export. Therefore, comparing HRES‐4 (grid + PV + SLB) against G‐PV quantifies the incremental effect of storage alone, whereas comparing G‐PV against the grid‐only case quantifies the effect of PV alone.

**TABLE 1:** Summary of system components for the different HRES configurations.

System	PV	Grid	Converter	Battery
Grid	No	Yes	No	No
G‐PV	Yes	Yes	Yes	No
HRES‐1	No	Yes	Yes	New
HRES‐2	No	Yes	Yes	Used
HRES‐3	Yes	Yes	Yes	New
HRES‐4	Yes	Yes	Yes	Used

### 2.2 Second‐Life Battery Modeling in HOMER

Recently, the integration of battery containers in real‐life applications with different RE systems has emerged as a promising solution for enhancing energy reliability and improving the power balance within the system [35, 36, 37]. However, most existing studies primarily focus on the feasibility of integrating new batteries, which typically exhibit higher energy storage capacities and better economic performance [38, 39]. Therefore, it is essential to evaluate the techno‐economic, environmental, and storage performances of SLBs. In this context, SLBs have recently gained significant attention as an emerging trend, attracting considerable interest from the research community [40]. However, several operational criteria must be considered when using this method. Batteries repurposed for SLB applications exhibit heterogeneous characteristics, including variations in the state of charge (SOC), degradation levels, and operational history [41]. These disparities necessitate the development of advanced control strategies to ensure effective integration in both series and parallel configurations [42].

The SOC is a fundamental variable for managing charging, discharging, balancing, and health monitoring processes [43]. The behavior of SOC in batteries with diverse characteristics has been extensively investigated in the literature because of its critical role in the performance of energy storage systems [44, 45]. Accordingly, the scaled SOC must be optimized at the battery container level to evaluate the feasibility of SLB‐based storage systems using the HOMER software. To determine this parameter, Equation (3) is employed to calculate the optimized scaled SOC, which can be expressed as follows [46]:

(3) $$SOC_{scaled}\left(k\right)=\frac{SOC_{pack}\left(k\right)-\;SOC_{min}}{SOC_{max}-\;SOC_{min}}\;$$

where *SOC_min_* and *SOC_max_* represent the lower and upper bounds of the operational window of the lithium battery, respectively. The *SOC_pack_* denotes the pack‐level SOC for 𝑛 batteries and can be defined as follows [45]:

(4) $$SOC_{pack}\left(k\right)=\frac{\sum_{i=1}^{n}Q_{max,i}.SOC_{i}\left(k\right)}{\sum_{i=1}^{n}Q_{max,i}}\;$$

where *k* represents the discrete time index, *Q*_*max*,*i*_ denotes the maximum capacity of the 𝑖‐th battery, and *SOC_i_* is the initial SOC of the 𝑖‐th battery, which can be estimated as follows [45, 47]

(5) $$SOC_{i}\;\left(k\right)=SOC_{i}\;\left(k-\Delta t\right)-\frac{P_{bat\left(t\right)\times \Delta t}}{B_{nom}}\;$$

where Δt is the sampling period in seconds, *P_bat_* (*t*) indicates the power variation at a particular time and, *B_nom_* refers to the battery's nominal storage capacity, a key parameter in system design and analysis.

LCOS is one of the key economic metrics considered in this study to assess the economic feasibility of using SLB systems. It is used to evaluate the equivalent cost of storing and delivering energy over the entire lifetime of the SLB system. This metric can be calculated as follows [48]:

(6) $$LCOS=\frac{\sum_{t=1}^{N}\frac{C_{t}}{{\left(1+r\right)}^{t}}}{\sum_{t=1}^{N}\frac{E_{t}}{{\left(1+r\right)}^{t}}}\;$$

where *C_t_* denotes the annual cost of the storage system in year *t*, *E_t_* ​ represents the energy discharged in year *t*, *r* is the discount rate, and *N* is the system lifetime.

Several key assumptions were adopted in this study to evaluate the performance of the SLB system. The initial SoH was assumed to be uniform across all cells at 80%, reflecting the typical condition of second‐life batteries at the beginning of their deployment. This is a simplifying assumption, and it is important to state plainly what it captures and does not capture. This value depends on the prior operating conditions and service life before the batteries are removed from electric vehicles. The battery service life was assumed to range from 10 to 15 years, depending on the operating conditions, throughput rate, and environmental factors. The cycle life was modeled as a function of the depth of discharge, with deeper cycles resulting in accelerated degradation. This degradation behavior was implemented in the HOMER simulation environment. In addition, a sensitivity analysis of this parameter was conducted to assess the feasibility of the SLB system under various operating conditions.

The assumption of uniform SoH represents a deliberate simplification of real second‐life battery packs. In practice, retired EV cells exhibit significant variability in residual capacity, internal resistance, and degradation owing to differences in their operating history. In this study, Equations (3)–(5) aggregate individual cell behavior into an equivalent pack‐level SOC, whereas HOMER Pro models the container as a single homogeneous storage unit with uniform capacity, efficiency, and degradation characteristics. Consequently, the per‐cell notation in Equations (4) and (5) describes the aggregation process rather than explicit cell‐level dynamics.

This assumption is consistent with the system‐level scope of HOMER Pro, which does not support cell‐resolved modelling. The objective of this study was to evaluate the techno‐economic and environmental feasibility of containerized second‐life batteries at the system scale, where aggregate storage capacity, annual energy throughput, and acquisition cost are the dominant performance indicators. Accordingly, the equivalent‐pack representation is an appropriate and widely adopted abstraction for HOMER‐based techno‐economic analyses. Nevertheless, this simplification introduces optimistic performance estimates. In heterogeneous packs, weaker cells constrain the usable operating window, whereas cell‐to‐cell resistance mismatch increases power losses, thermal stress, and degradation. Consequently, the usable capacity, round‐trip efficiency, and service life are expected to be lower than those predicted by the homogeneous model. Therefore, the storage performance reported in Section 3.2, particularly the estimated SLB lifetime of 11.8 years, should be interpreted as an upper‐bound estimate.

### 2.3 Optimization Framework

**FIGURE 2:**
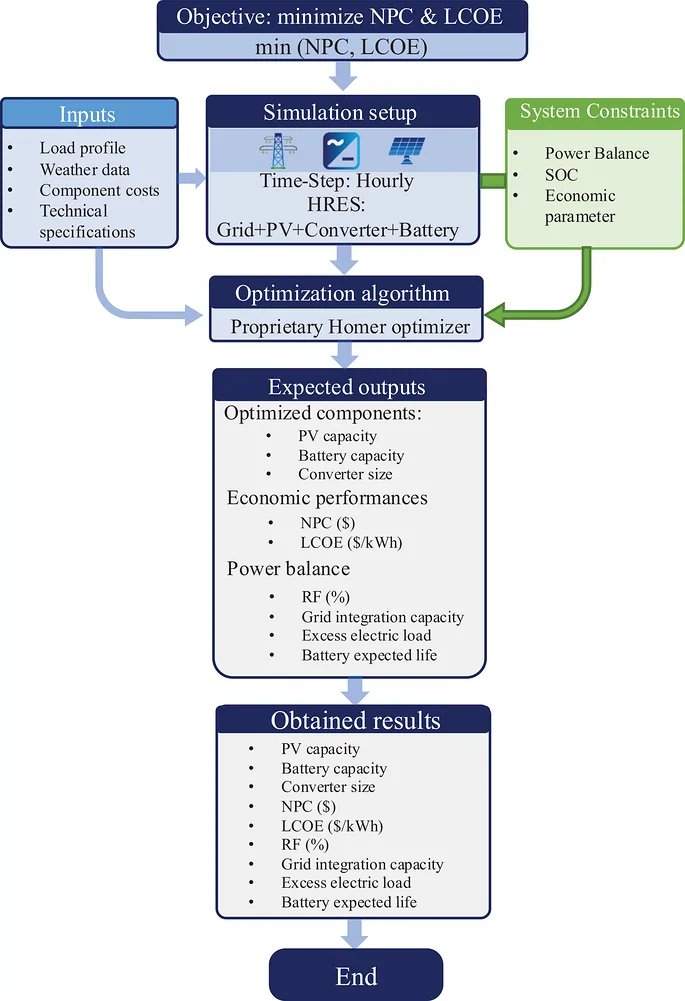
Optimization framework for the proposed HRES.

HOMER software is an advanced simulation tool for energy systems that can be used for various purposes, including simulation, optimization, and sensitivity analysis, to investigate the feasibility of the proposed HRES relative to a baseline system. This software allows the determination of the most cost‐effective and reliable mix of energy resources for both on‐grid and off‐grid projects. In this study, the optimization of the proposed HRES was based on the primary objective of minimizing both the net present cost (NPC) and LCOE, as illustrated in Figure 2. The objective function can be expressed as follows:

(7) $$\min\left(NPC,LCOE\right)$$

where the NPC accounts for all lifecycle costs related to system operation, while the LCOE represents the average cost of electricity generation per unit of energy produced. Both costs can be determined using Equations (8) and (9), respectively [49]:

(8) $$\mathrm{NPC}\;=\sum_{i}^{N}\frac{NC_{i}}{{\left(1+R\right)}^{i}}-\mathrm{TInvC}$$

(9) $$\mathrm{LCOE}\;=\frac{\mathrm{TAC}}{E_{y}}\;$$

where *TAC* represents the total annualized cost, *TInvC* refers to the overall investment cost, *CRF* denotes the capital recovery factor, and *NC_i_* corresponds to the net cash flow in year *i*.

The simulation was conducted at an hourly time resolution, equivalent to 8760 h per year, to capture temporal variations in solar irradiance and load demand. This simulation was also used to determine the optimal component sizes of the system. Several constraints were applied to this optimization to identify the most suitable, feasible, and low‐cost configuration. In this optimization, the main constraints considered include power balance, battery SOC, and economic parameters such as inflation and the nominal discount rate. By considering these constraints, HOMER uses a proprietary optimization algorithm to identify the most economically and technically suitable combination of components with sizes optimized within predefined ranges. Therefore, the optimized HRES system was ranked based on the obtained system sizes and its economic and environmental performance. It should be noted that the economic analysis and capital investment of the different system configurations take into account the cost of the used battery.

System operation is governed by the load‐following dispatch strategy, which determines battery charging behavior and the allocation of benefits between PV generation and second‐life battery storage. Under this strategy, renewable generation is first used to satisfy the instantaneous load, with surplus electricity directed to battery charging and subsequently exported to the grid once the battery reaches its operating limit. The battery operates within an SOC range of 40%–95%, corresponding to a usable capacity of 538 kWh from the 896 kWh containerized battery, thereby limiting degradation. Power balance is enforced at every hourly time step. The technical and economic input parameters supplied to the HOMER Pro model are summarized in Table 2.

**TABLE 2:** Technical and economic input parameters supplied to the HOMER Pro model.

Parameter	Value
Time resolution	1 h (8760 steps/yr)
Project lifetime	25 yr
Nominal discount rate	3.2%
Expected inflation rate	2.2%
Max capacity shortage	95%
PV ARRAY	
Module type / rating	Monocrystalline, 310 W
Module efficiency	19%
Capital cost	$270/kWp
Replacement cost	$270/kWp
O&M cost	$5/year
Lifetime	25
Derating factor	80%
Temperature coefficient	−0.38%/^0^C
POWER CONVERTER	
Inverter efficiency	95%
Rectifier efficiency	95%
Capital / replacement cost	$300/kWp
O&M cost	$5/year
Lifetime	15
BATTERY STORAGE	
Chemistry / configuration	LiFePO_4_; 700 cells; 25 strings x 28 cells at 12.8 V
Nominal / usable capacity	896 kWh / 538 kWh
Minimum state of charge	40%
Capital cost, new battery	$300/unit
Capital cost, second‐life battery	$150/unit
Replacement cost (new / SLB)	$260/unit
O&M cost	$130/unit
Initial state of health (SLB)	80%
Baseline SLB throughput	3584
GRID AND EMISSIONS	
Grid purchase price	$0.052/kWh
Grid sellback price	$0.05/kWh
Grid emission factor (F)	0.862 kg CO_2_/kWh
Carbon tax rate	$20/ton
Carbon credit rate	$25/ton

HOMER Pro optimizes each configuration by selecting component sizes that minimize the NPC within predefined search ranges. The PV array was optimized over a search range of 500–1500 kW, while the converter capacity was optimized automatically according to the system requirements, converging to 1000 kW PV and 499 and 492 kW converters for HRES‐3 and HRES‐4, respectively. The second‐life battery was modelled as a fixed 896 kWh container rather than a continuously scalable asset, consistent with its physical design. Storage‐free reference systems were optimized using the same search methodology to ensure a fair and unbiased comparison.

The economic advantage of HRES‐4 arises primarily from the substantially lower acquisition cost of second‐life batteries rather than differences in operational strategy. Although the new and second‐life batteries exhibit different SOC characteristics, energy throughput, and service life, both systems employ identical PV capacities and comparable operating conditions. Therefore, the storage‐level performance metrics should be interpreted separately, while the system‐level indicators presented in Section 3.3 provide the appropriate basis for comparing the overall techno‐economic performance of the evaluated configurations.

### 2.4 Environmental Impact

One of the main objectives of using SLBs is to reduce the environmental impact of a microgrid. This section presents the mathematical equations used to evaluate the environmental benefits of integrating SLBs. The carbon emissions of the proposed system were assessed over the entire life cycle, encompassing three phases: manufacturing and installation, operation (production), and recycling. Therefore, the total carbon emissions can be estimated as follows [50]:

(10) $$PE_{i}=F_{i}\;.\left(CExC+P_{Grid}\right)$$

The mitigated emissions (*PM_i_*) of the proposed system is related to the annual power generated by the PV system (*E*_*y*, *PV*_) where and can be calculated as follows [50]:

(11) $$PM_{i}=F_{i}\;.E_{y,PV}$$

where *F_i_* denotes the pollutant emission factor associated with electricity generation from the coal power plant, *P_Grid_* denote the grid supply, and the *CExC* represents the equivalent cumulative resource consumption and can be calculated as follows [51]:

(12) $$CExC=\frac{\mathrm{TCC}}{C_{el}}\;$$

where: *C_el_* denotes the cost of electricity in Malaysia under the commercial (business) tariff.

The scope of this assessment is narrower than that of a conventional process‐based life‐cycle assessment. The system boundary primarily considers the operational phase, including the emissions associated with electricity imported from the Malaysian grid over the 25‐year project lifetime and the emissions displaced by on‐site PV generation. The functional unit is the annual electricity demand of the residential community described in Section 2.1 (603.86 MWh/year), evaluated over the project lifetime. The manufacturing of new battery cells, refurbishment of retired batteries, transportation from the point of retirement to the deployment site, and end‐of‐life treatment are not explicitly modelled as separate life‐cycle inventory processes or assigned component‐specific embodied‐carbon emission factors. Instead, these lifecycle activities are implicitly represented through the cumulative exergy consumption (CExC) term, which is derived from the total capital cost (TCC) of the system. The TCC represents the cumulative economic investment over the project lifetime, including the initial capital expenditure, together with replacement, operation, and maintenance costs. Equation (12) converts this lifetime expenditure into an equivalent quantity of grid electricity by dividing the total cost by the commercial electricity tariff. Consequently, the CExC is expressed as a cost‐equivalent energy proxy (MWh) representing the total exergy associated with the resources required to construct, operate, maintain, and replace the system throughout its lifetime, rather than a direct inventory of embodied energy or embodied carbon. When the Malaysian grid emission factor is subsequently applied in Equation (10), it implicitly assumes that each dollar of expenditure results in emissions equivalent to those associated with purchasing grid electricity, regardless of differences in the carbon intensity of individual system components. The implications and limitations of this assumption are discussed in Section 3.7. Therefore, the total carbon emissions are estimated from the grid electricity consumed during operation together with the CExC term, while the avoided emissions are determined from the electricity generated by the PV system, as expressed in Equations (10)–(12).

After quantifying the emitted and avoided CO_2_ emissions, the carbon credit $\left(CRI_{CO_{2}}\right)$ and carbon tax $\left(CT_{CO_{2}}\right)$ are evaluated to comprehensively assess the techno‐economic and environmental performance of the proposed system. These parameters are calculated as follows [51]:

(13) $$CRI_{CO_{2}}=cri_{CO_{2}}.PM_{CO_{2}}$$

(14) $$CT_{CO_{2}}=ct_{CO_{2}}.PE_{CO_{2}}$$

## 3 Results and Discussion

The simulation results obtained with HOMER identified six feasible configurations, comprising two reference cases (grid and G‐PV) and four HRES configurations. The performance of the optimal system is compared and presented in this section. These results highlight the benefits of integrating SLBs into various HRES configurations.

### 3.1 System Electrical Performances

#### 3.1.1 Overall Electrical Output

**FIGURE 3:**
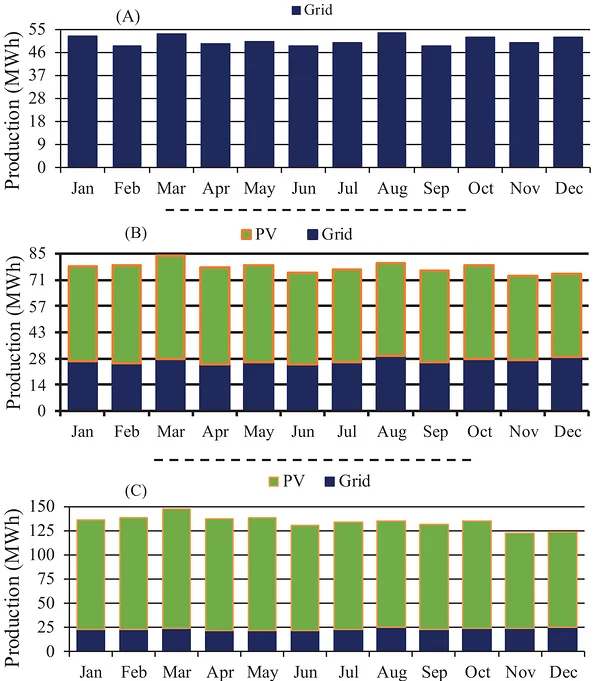
Power production of different HRES systems: (A) Grid, HRES‐1, and HRES‐2; (B) G‐PV; (C) HRES‐3 and HRES‐4.

The electrical performance simulation outputs were analyzed to evaluate the power‐sharing contribution of each system component across different configurations. The results are shown in Figure 3.

Monthly electricity production across different system configurations, as shown in Figure 3. Figure 3A shows the monthly grid output for the conventional system, HRES‐1, and HRES‐2. The results indicate that the grid contributes 100% of the energy in these three systems, with an annual power output of 603 856 kWh. Additionally, the monthly electricity supply remains relatively stable, particularly in March, August, and December. The inclusion of energy storage systems does not significantly affect the overall energy consumption, indicating that the grid provides a consistent, dispatchable power supply.

Figure 3B presents the monthly electricity production of the G‐PV reference case, in which the PV array is operated without any battery storage. Under this configuration, the optimized PV capacity settles at a considerably smaller value than in HRES‐3 and HRES‐4, and the annual PV output is limited to 602.09 MWh, i.e., only about 45% of the 1337.99 MWh generated in the battery‐equipped configurations. Without storage to absorb excess daytime generation, surplus PV electricity is curtailed, requiring the grid to supply 8133.25 MWh over the 25‐year project lifetime (approximately 325 MWh/year) and resulting in a renewable fraction of 63.2%. This reference case highlights that PV generation alone provides only limited reductions in grid dependency, whereas the full benefits of the PV system are realized when battery storage enables surplus energy to be shifted to periods of higher demand.

Figure 3C illustrates the power‐sharing contribution of the PV system throughout the year for HRES‐3 and HRES‐4. The results show that the total system output in both configurations increases significantly, reaching approximately 300% of the baseline system's output. Furthermore, the PV system accounts for about 83.1% of the total system output, with an annual energy production of 1337.99 MWh across both configurations. The grid supplies the remaining 16.9%, corresponding to 272 873 kWh and 272 633 kWh for HRES‐3 and HRES‐4, respectively. This difference is attributed to variations in the initial SOC of the energy storage systems in the two configurations.

The results indicate that the grid‐only systems, HRES‐1 and HRES‐2, exhibit nearly identical annual grid energy purchases of approximately 603 MWh, with no energy exported to the grid. In contrast, HRES‐3 and HRES‐4 significantly reduced grid purchases to approximately 273 MWh, representing a reduction of more than 54.73% owing to the integration of the PV system. Moreover, HRES‐3 and HRES‐4 demonstrate substantial energy export to the grid, exceeding 820 MWh annually. In addition, excess electricity generation is estimated at approximately 109.9 MWh and 115.98 MWh for HRES‐3 and HRES‐4, respectively, exceeding the load demand by 6.82% and 7.2%, respectively. These findings indicate that integrating PV with energy storage systems transforms the system from a grid‐dependent configuration into a net energy exporter.

#### 3.1.2 PV System Operating Behaviors

**FIGURE 4:**
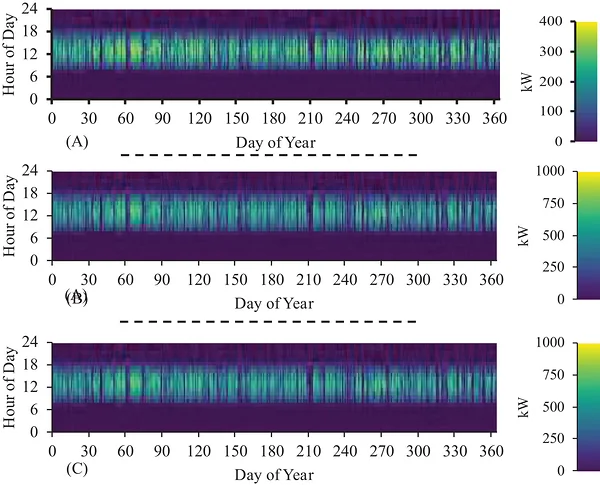
PV system operating behaviors (A) G‐PV, (B) HRES‐3, and (C) HRES‐4.

The optimized rated capacity of the PV system in HRES‐3 and HRES‐4 is 1000 kW, whereas the storage‐free G‐PV reference case is optimized at a markedly lower capacity because any generation beyond the instantaneous load can only be exported or curtailed. The operating behaviors of HRES‐3 and HRES‐4 are therefore expected to be similar to one another but distinct from the G‐PV case, as presented in Figure 4.

Figure 4 illustrates the operating behaviors of the PV system on both daily and annual timescales. The results show that PV generation occurs between 06:00 and 18:00, with peak energy production around midday. This reflects the typical solar availability profile in Malaysia. The results also indicate seasonal variability, with higher energy production observed during the mid‐year period (approximately days 100–250) and lower production at the beginning and end of the year. This trend corresponds to variations in solar irradiance throughout the year. In addition, short‐term fluctuations in the PV power output were evident, reflecting the impact of weather variability, particularly transient cloud cover conditions in Malaysia. Furthermore, the results show that the specific yield is 1338 kWh/kW, with a PV penetration level of 222%.

The G‐PV reference case (Figure 4A) exhibits the same diurnal and seasonal generation profile as HRES‐3 and HRES‐4 because all configurations were subjected to identical solar irradiance and ambient temperature conditions. However, the annual PV generation was limited to 602.09 MWh, corresponding to a PV penetration of approximately 100%, as the optimal PV capacity was constrained by the instantaneous load demand in the absence of energy storage. This comparison clearly isolates the operational role of battery storage. Although the temporal characteristics of PV generation are determined solely by the available solar resource and are therefore independent of storage, the economically optimal PV capacity depends on the system's ability to capture and utilize surplus generation. Without battery storage, excess electricity produced during periods of high solar irradiance cannot be shifted to later demand periods and is consequently curtailed, prompting the optimization algorithm to limit the installed PV capacity. In contrast, the integration of the SLB bank enables surplus PV generation to be stored and dispatched when required, substantially reducing curtailment and supporting the economically optimal deployment of a 1000 kW PV array while maintaining a comparable specific energy yield. These results demonstrate that the primary contribution of the SLB system is not to increase PV conversion efficiency, but to enhance RE utilization by improving temporal matching between electricity generation and demand, thereby enabling significantly higher PV penetration within the hybrid energy system.

Overall, these results indicate that including different energy storage configurations in both HRES systems improves the effective power output compared to both baseline systems, and that the improvement over the G‐PV baseline is attributable to storage rather than to the PV resource itself. On the other hand, the results confirm that the initial SOC of the batteries does not significantly affect the system's power output; rather, it primarily affects the economic and environmental performance of these systems. In this context, the following subsection evaluates these aspects in greater detail.

#### 3.1.3 System Converter Operating Behaviors

**FIGURE 5:**
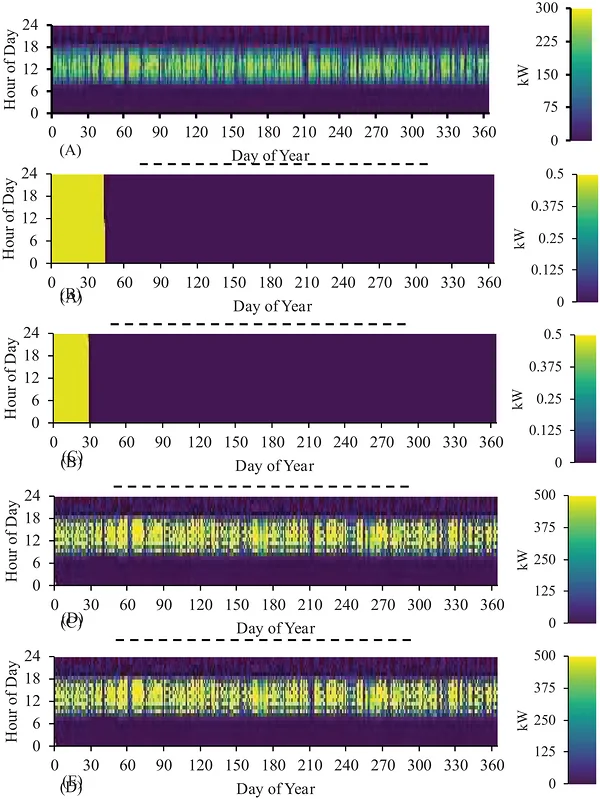
System converter operating behaviors (A) G‐PV, (B) HRES‐1, (C) HRES‐2, (D) HRES‐3, (E) HRES‐4.

The converter is a critical component of system configurations that incorporate battery storage. In this section, the operational behavior of the converter across different configurations is evaluated, and the obtained results are presented in Figure 5.

As illustrated in Figure 5, the different system configurations exhibit distinct converter operating behaviors. This variation is primarily attributed to differences in system architecture, including the components connected to the DC and AC buses, as well as variations in power output. For instance, the converter operation in HRES‐1 and HRES‐2 is limited and primarily concentrated at the beginning of the year, specifically within the first 30–50 days. During the remainder of the year, the converter remains largely inactive. The results indicate that the maximum operating power was approximately 0.48 kW, reflecting minimal utilization owing to the absence of a PV system in these configurations (see Figure 5B,C). The G‐PV reference case, by contrast, requires a converter rated at 269 kW that operates for 4358 h/yr (Figure 5A), confirming that most of the converter duty in a PV‐based system is created by the PV array rather than by the battery. In contrast, the converters in HRES‐3 and HRES‐4 operate continuously throughout the year, with operating patterns closely aligned with PV power generation. The results show that the converter reaches a peak power of approximately 500 kW during daylight hours, which corresponds to the PV system's operational period. This enables active DC–AC conversion and facilitates energy exchange between the DC and AC buses (see Figure 5D,E). To further illustrate the converter performance across different configurations, Table 3 presents a comparative analysis of the system converters.

**TABLE 3:** System converter operating performances for the different configurations.

Quantity	G‐PV	HRES‐1	HRES‐2	HRES‐3	HRES‐4
Capacity (kW)	269	0.480	0.480	499	492
Mean Output (kW)	63.9	0.0571	0.0571	132	132
Maximum Output (kW)	269	0.480	0.480	499	492
Capacity Factor (%)	23.8	11.9	11.9	26.5	26.8
Hours of Operation (hr/yr)	4358	1,044	696	4708	4550
Energy Out (MWh/yr)	559.78	0.500	0.334	1159.25	1153.04
Energy In (kWh/yr)	589.24	0.527	0.351	1220.26	1213.72
Losses (kWh/yr)	29462	26.3	17.6	61013	60686

Table 3 confirms that different system architectures exhibit distinct performance characteristics. For instance, the converters in HRES‐1 and HRES‐2 show a low mean output of 0.0571 kW and limited annual operating hours of 1044 and 696 h, respectively. This is primarily due to their low‐rated capacity (i.e., 0.48 MW). The results also indicate a low capacity factor of 11.9%, reflecting the limited utilization of the converter across both configurations. This behavior can be explained by the direct supply of electricity from the grid to the load, while the storage system operates with minimal conversion requirements. In contrast, the integration of the PV system in HRES‐3 and HRES‐4 results in substantially higher converter capacities of 499 and 492 kW, respectively. The peak output reaches the rated capacity, with a mean converter output of approximately 132 kW for both configurations. Furthermore, the annual electrical energy processed by the converter is approximately 1159.25 MWh for HRES‐3 and 1153.04 MWh for HRES‐4, with annual operating durations of 4708 and 4550 h, respectively. This indicates continuous daily operation driven by PV generation. The results also show that the converters in HRES‐3 and HRES‐4 achieve higher capacity factors of 26.5% and 26.8%, respectively, with energy losses of approximately 60 MWh/year. These losses are expected given the system's high energy throughput.

The performance of the HRES‐3 and HRES‐4 systems against the G‐PV reference case enabled the individual contributions of the PV array and SLB system to be distinguished. Compared with the grid‐only and storage‐only configurations, the integration of PV alone increased the optimal converter capacity from 0.48 to 269 kW, raised the annual energy processed from less than 1 to 559.78 MWh/year, and improved the converter capacity factor to 23.8%, reflecting the additional power conversion required for PV generation. The subsequent integration of the SLB system further increased the optimal converter capacity by approximately 83%, from 269 to 492 kW, while nearly doubling the annual energy throughput from 559.78 to 1153.04 MWh/year. Simultaneously, the converter capacity factor increases from 23.8% to 26.8%, and the annual operating duration extends from 4,358 h/year to 4,550 h/year. These improvements demonstrate that the primary contribution of battery storage is to enhance converter utilization by shifting excess PV generation to periods without solar production, thereby maintaining converter loading beyond daylight hours, rather than restricting operation to periods of PV generation. As expected, the increased energy throughput resulted in proportionally higher converter losses, rising from 29.5 MWh/year in the G‐PV configuration to approximately 60 MWh/year in HRES‐3 and HRES‐4, consistent with the substantially greater volume of energy processed.

### 3.2 Energy Storage System

**FIGURE 6:**
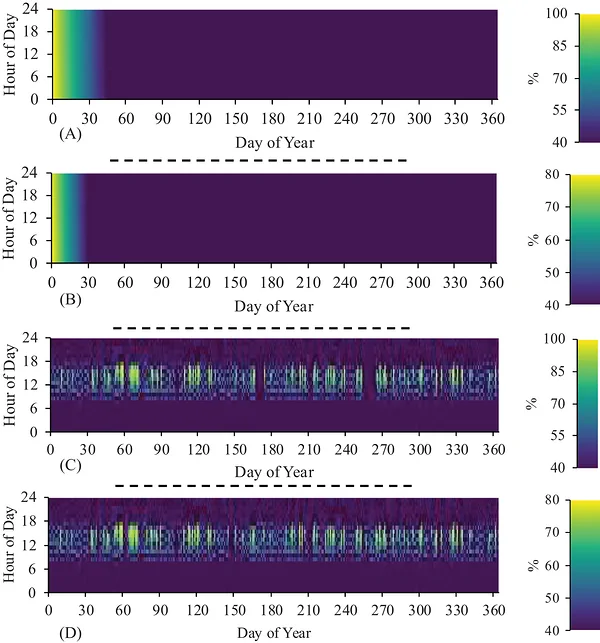
Battery SOC for (A) HRES‐1, (B) HRES‐2, (C) HRES‐3, (D) HRES‐4.

This study focuses mainly on the benefits of using the SLB in the HRES to improve the energy feasibility of the system. This section analyzes the energy storage system performance, and the obtained results are presented in Figure 6.

Figure 6 shows the temporal evolution of the battery SOC for the different system configurations. A clear distinction can be observed between grid‐dependent (i.e., HRES‐1 and HRES‐2) and renewable‐integrated systems (i.e., HRES‐3 and HRES‐4). For instance, the SOC profiles of the storage systems in HRES‐1 and HRES‐2 show high charging levels approaching 100% during the initial period of operation, followed by a rapid decline after a few days. This trend is consistent with the operating behavior observed for the converters. These results indicate that the battery systems are used only minimally beyond the initial operating period, reflecting the dominance of the grid supply in both configurations. Consequently, the storage systems remained largely inactive. In contrast, the SOC profiles of the storage systems in HRES‐3 and HRES‐4 exhibited continuous and dynamic fluctuations throughout the year. The SOC trends closely followed both daily and seasonal variations in PV power generation. Charging primarily occurred during the daytime, whereas discharging occurred at night when PV generation was unavailable. This behavior reflects the active role of the storage systems in energy balancing within the HRES, with frequent charge–discharge cycles demonstrating effective battery utilization in both configurations. A slightly smoother SOC trace is discernible for HRES‐4 than for HRES‐3. This should not be over‐interpreted: the two configurations operate the same PV array against the same load under the same dispatch strategy; thus, no difference in energy management arises from the battery chemistry, and none is claimed here. The visual difference is consistent with the marginally larger surplus absorbed by HRES‐4, which is reflected in its slightly higher annual throughput reported in Table 4, rather than with any superior control behavior attributable to the use of second‐life cells. The SOC range in both configurations remained within the operational limits, indicating appropriate system sizing and effective control strategies.

**TABLE 4:** Storage system operating performances for the different configurations.

Quantity	HRES‐1	HRES‐2	HRES‐3	HRES‐4
Autonomy (hr)	7.80	7.80	7.80	7.80
Storage Wear Cost ($/kWh)	0.00415	0.00427	0.00415	0.00427
Nominal Capacity (kWh)	896	896	896	896
Usable Nominal Capacity (kWh)	538	538	538	538
Expected Life (yr)	ND	ND	14.6	11.8
Energy Out (kWh/yr)	527	351	201776	207651
Storage Depletion (kWh/yr)	538	358	538	358
Losses (kWh/yr)	10.9	7.24	8,396	8,645
Annual Throughput (kWh/yr)	538	358	205,937	211,933
Annual EFCs (1/yr)	0.600	0.400	230	237

Table 4 compares the operational performance of the energy storage systems across the different HRES configurations and confirms the behaviors observed in Figure 6. The results indicate that the optimal storage capacity is approximately 7.8 h for all configurations, with a nominal capacity of 896 kWh and a usable capacity of 538 kWh. This consistency is attributed to the standardized sizing of the containerized storage system.

The results show that in both HRES‐1 and HRES‐2, the energy storage systems exhibited very limited utilization, with annual energy output ≤ 527 kWh, annual throughput ≤ 538 kWh, and EFCs in the range of 0.4–0.6 cycles per year. This reflects the minimal operational role of the storage system in these configurations. In contrast, integrating the PV system into HRES‐3 and HRES‐4 leads to significantly increased storage activity, with annual energy output exceeding 200 MWh, annual throughput exceeding 200 MWh/year, and more than 230 equivalent full cycles per year.

Notably, HRES‐4 demonstrated the highest storage utilization, with an annual energy output of 207.65 MWh and approximately 237 equivalent full cycles per year. Although HRES‐4 exhibited a slightly higher storage wear cost (0.00427 $/kWh) and a shorter lifetime (11.8 years), which was approximately 2.8 years lower than that of HRES‐3, this behavior reflects the intensified utilization associated with second‐life batteries. The comparability of HRES‐3 and HRES‐4 on these annual indicators requires mechanistic explanation rather than assertion, since it is not an obvious outcome: the second‐life bank has a service life 2.8 years shorter and a marginally higher wear cost, yet it returns a marginally higher annual output. Taken together, these findings confirm that second‐life lithium‐ion batteries can provide a technically viable and economically attractive solution when integrated with PV systems. Furthermore, the results support the feasibility of extending the lifecycle of lithium‐ion batteries for secondary applications rather than immediate recycling.

The similar annual throughput and cycling performance of HRES‐3 and HRES‐4 arise from the system architecture rather than the battery condition. Both configurations employ the same 1000 kW PV array, operate under identical irradiance and load profiles, and use the same containerized battery with a nominal capacity of 896 kWh and a usable capacity of 538 kWh. Consequently, both storage systems receive the same surplus PV energy and perform the same charge–discharge duty. The battery therefore follows the system demand rather than determining it. As a result, the annual throughput (205.9 and 211.9 MWh/year) and equivalent full cycles (230 and 237 cycles/year) are governed primarily by the available PV surplus rather than the battery state of health.

This interpretation is supported by the storage performance metrics reported in Table 4. The loss fraction remains nearly identical in both systems (approximately 4.1%), indicating that the lower initial state of health of the second‐life battery does not significantly reduce the pack‐level round‐trip efficiency within the adopted 40%–95% SOC operating window. Likewise, the energy delivered per equivalent full cycle is almost identical, confirming that both batteries experience comparable cycling depth and dispatch behavior. The identical storage autonomy (7.8 h) further reflects the standardized container design.

The shorter expected lifetime of HRES‐4 (11.8 years vs. 14.6 years) does not indicate inferior annual operating performance. Instead, the same annual duty consumes a larger fraction of the remaining lifetime of a battery that begins operation at 80% state of health. Consequently, although both systems exhibit comparable annual storage performance, they differ in lifetime energy delivery. Based on the projected service life, HRES‐3 delivers approximately 3007 MWh over its lifetime, whereas HRES‐4 delivers approximately 2501 MWh, representing approximately 17% lower lifetime energy throughput. Nevertheless, the substantially lower acquisition cost of the second‐life battery more than compensates for its shorter lifetime, resulting in the lowest net present cost among all evaluated configurations.

### 3.3 Techno‐Economic Performance Analysis

The economic performance of the different configurations is analyzed in this section. The results include cumulative cash flows and key economic indicators, which are presented in Tables 5 and 6, respectively.

**TABLE 5:** Cumulative cash flow for the different configuration.

Operating year	Grid ($)	G‐PV ($)	HRES‐1 ($)	HRES‐2 ($)	HRES‐3 ($)	HRES‐4 ($)
0	0	202088	245143.90	105143.90	664662.50	522551.90
5	164199.60	233193	426202.30	286247.70	579044.90	438436.20
10	320551.80	262812	598607.60	458696.10	497519.20	358340.60
15	469431.60	360592	762897.50	623027.10	559719.20	418814.70
20	611196	387447	919217.20	779386.10	485799.90	346192.10
25	746185.20	391991	1058482	920044.50	373580.90	239565.30

**TABLE 6:** Economic performance for the different configurations.

Quantity	Grid	G‐PV	HRES‐1	HRES‐2	HRES‐3	HRES‐4
CAPEX ($)	0	202088	245144	105144	664663	522552
Operating cost ($/yr)	33815.94	8606	36859	36930	−13191	−12825
LCOE ($/kWh)	0.056	0.0201	0.0794	0.0794	0.0118	0.00762
LCOS ($/kWh)	0	0	23.73	37.61	0.069	0.072
Annual worth ($/yr)	0	16052	0	0	31039	30838
Return on investment (%)	0	8.5	0	0	11.5	13.4
Simple payback (yr)	ND	7.37	ND	ND	7.59	6.75

Table 5 illustrates the cumulative cash flow over a 25‐year project lifetime. It can be observed that the different system configurations exhibit distinct cost trajectories, and that the G‐PV reference case provides the appropriate benchmark for separating the value of PV from the value of second‐life storage. The Grid, HRES‐1, and HRES‐2 configurations are characterized by low initial investments but progressively increasing long‐term costs. The grid‐only system requires no initial capital investment; however, its cumulative cost increases continuously, reaching approximately $746.19K after 25 years due to its complete dependence on grid electricity. Meanwhile, HRES‐1 and HRES‐2 require moderate initial investments of $245K and $105K, respectively, but result in the highest lifecycle costs of approximately $1.06M and $920K. This outcome can be attributed to the integration of energy storage systems without renewable generation, leading to continued reliance on grid electricity and limited operational cost savings. Therefore, storage‐only configurations combined with grid supply are economically unfavorable, as they increase capital expenditure without significantly reducing operating costs. In contrast, HRES‐3 and HRES‐4 require higher initial investments but have lower lifecycle costs. HRES‐3 records an initial investment of approximately $664.66K, which is about 21% higher than HRES‐4's. Both configurations exhibit a declining trend in cumulative cost growth over time, reflecting their long‐term economic advantages. Furthermore, the cumulative costs of these two configurations increase at a significantly slower rate, indicating improved economic performance. Notably, HRES‐4 is ranked as the most economically feasible configuration, with an NPC of approximately $239.56K. This represents a reduction of approximately 68%, 77%, 74%, and 36% compared to the Grid, HRES‐1, HRES‐2, and HRES‐3 systems, respectively.

The G‐PV reference case represents an intermediate economic scenario between the grid‐dependent configurations (conventional, HRES‐1, and HRES‐2) and battery‐assisted systems (HRES‐3 and HRES‐4). It requires a relatively modest initial capital investment of $202.09K and accumulates a total lifetime cost of $391.99K over the 25‐year project horizon, representing a 47% reduction compared with the grid‐only configurations. This result indicates that a substantial proportion of the economic benefits achieved by HRES‐3 and HRES‐4 is attributable to PV generation and grid export, rather than battery storage alone. However, the integration of an SLB in HRES‐4 further reduces the cumulative lifetime cost to $239.57K, corresponding to an additional 39% reduction relative to the G‐PV configuration. This improvement is achieved despite HRES‐4 requiring an initial capital investment approximately 2.6 times greater than that of G‐PV, demonstrating that the additional storage investment is offset by its ability to capture surplus PV generation that would otherwise be curtailed and to dispatch this energy during periods of higher electricity value or demand. Consequently, the cumulative cost trajectory of HRES‐4 becomes noticeably flatter after approximately the tenth year of operation, reflecting the long‐term economic value of enhanced RE utilization. Importantly, this advantage is unique to the second‐life battery configuration. Although HRES‐3 employs the same PV capacity, its reliance on new batteries results in a cumulative lifetime cost of $373.58K, which is comparable to the $391.99K obtained for the storage‐free G‐PV case. These findings demonstrate that the economic value of battery storage is highly dependent on storage cost, with significant lifecycle savings realized only when low‐cost second‐life batteries are employed.

The superior economic feasibility of the proposed HRES‐4 configuration is clearly illustrated in Table 6, which compares the key economic indicators of the different system configurations. The results demonstrate a clear transition from cost‐intensive conventional systems to economically optimized renewable‐based systems, with the G‐PV case serving to attribute the transition between its PV and storage components. The Grid, HRES‐1, and HRES‐2 configurations exhibited relatively high operating costs, ranging from $33 815.94 to $36 930 per year, and higher LCOE values of $0.056 to $0.0794/kWh. Additionally, integrating storage systems into HRES‐1 and HRES‐2 increased the capital expenditure (CAPEX) to $245 144 and $105 144, respectively, without delivering significant economic benefits. The G‐PV reference case improves markedly on all three, with a CAPEX of $202 088, an operating cost of $8606/yr, an LCOE of $0.0201/kWh, an ROI of 8.5% and a payback of 7.37 years, which establishes that PV alone, without any battery, is already an attractive investment at this site. In contrast, HRES‐3 and HRES‐4 demonstrate substantially improved economic performance. In particular, HRES‐4 is identified as the most economically favorable configuration. It achieves the lowest LCOE of $0.00762/kWh and exhibits a negative operating cost of approximately $12 825/year, indicating net revenue generation. Although its CAPEX reaches $522 552, it is approximately 21.38% lower than that of HRES‐3. HRES‐4 delivers the best economic performance, achieving an ROI of 13.4% and a simple payback period of 6.75 years, compared with 11.5% and 7.59 years for HRES‐3. As both configurations employed the same PV capacity and had nearly identical operating costs, this improvement was primarily attributed to the lower acquisition cost of the SLB, which generated comparable annual savings from a smaller capital investment.

Compared with the G‐PV reference case, integrating the SLB increases the ROI from 8.5% to 13.4% and reduces the payback period from 7.37 to 6.75 years. It also lowers the LCOE by 62% (from $0.0201/kWh to $0.00762/kWh), converts the annual operating cost from +$8606/year to −$12 825/year, and nearly doubles the annual worth from $16 052/year to $30 838/year. These improvements result from the battery's ability to store surplus PV generation, reduce curtailment, and increase electricity exports. Although the G‐PV configuration requires lower initial capital, the SLB‐equipped system delivers superior lifecycle economic performance by enabling higher PV utilization and greater export revenue, while its cost‐effectiveness is reflected by the low LCOS. The results show that HRES‐3 achieves the lowest LCOS of $0.069/kWh, $0.003/kWh lower than HRES‐4. In contrast, HRES‐1 and HRES‐2 exhibit significantly higher LCOS values of $23.73/kWh and $37.61/kWh, respectively. This indicates that storage‐only configurations combined with grid supply reduce the overall economic viability of the system. No LCOS is reported for the Grid and G‐PV cases because they contain no storage; this is precisely why the G‐PV comparison, rather than the LCOS, is the correct test of whether storage earns its place in the system. Taken together, these findings demonstrate that integrating PV systems with second‐life batteries offers a cost‐effective, technically viable, and financially sustainable solution. The superior economic performance of HRES‐4 is driven by two separable contributions. The comparison of G‐PV with the grid‐only case shows that the incorporation of RE accounts for the larger share, reducing grid dependency and the LCOE by 64% on its own. The comparison of HRES‐4 with G‐PV shows that the second‐life battery accounts for the remainder, and does so by removing the absorption limit that caps PV deployment in the storage‐free case rather than by any intrinsic arbitrage margin. At the same time, the use of second‐life lithium‐ion batteries lowers capital costs while maintaining strong long‐term performance, despite their reduced lifespan. Moreover, this approach extends battery lifecycle through secondary applications, offering an economically attractive alternative to immediate recycling and contributing to broader environmental sustainability objectives.

The exceptionally low LCOE values and negative operating costs of HRES‐3 and HRES‐4 result from substantial electricity exports rather than unusually low system costs. The optimized 1 MW PV array generates 1337.99 MWh/year against an annual load of 603.86 MWh, exporting more than 820 MWh/year (61% of total PV generation). Revenue from these exports exceeds the combined costs of grid purchases and system maintenance, resulting in negative annual operating costs of −$13 191/year for HRES‐3 and −$12 825/year for HRES‐4, indicating net operating revenue rather than zero operating expenses.

The same mechanism produces the low LCOE values. In HOMER, export revenue is deducted from the annualized system cost before calculating the LCOE. Consequently, when export revenue nearly offsets annual costs, the residual annualized cost becomes very small, yielding LCOE values of $0.0118/kWh and $0.00762/kWh for HRES‐3 and HRES‐4, respectively. Under these conditions, LCOE becomes highly sensitive to small variations in net annualized cost and is therefore less suitable for comparing exporting systems. Annual worth provides a more robust economic indicator, with HRES‐3 and HRES‐4 generating $31 039/year and $30 838/year, respectively, despite their different capital costs.

These economic results are inherently dependent on the electricity export tariff. Because exported electricity exceeds grid purchases, changes in the sellback tariff directly affect the NPC, LCOE, operating cost, and payback period. The relative ranking of HRES‐3 and HRES‐4 remains robust because both configurations export nearly identical amounts of electricity, preserving the economic advantage of the lower‐cost second‐life battery.

The economic results demonstrate that PV generation is the primary driver of cost reduction, whereas second‐life batteries maximize the economic value of generated RE. The G‐PV configuration reduces operating costs and LCOE by 74.55% and 64.11%, respectively, through reduced grid electricity purchases. In contrast, HRES‐1 (new battery) and HRES‐2 (second‐life battery) increase operating costs (8.99%–9.20%) and LCOE (41.79%) because, without PV, the batteries only shift grid electricity and cannot generate additional value. However, when integrated with PV (HRES‐3 and HRES‐4), storage enables surplus solar energy to be stored and utilized or exported more effectively, reducing operating costs by over 137% and lowering LCOE by 78.93% and 86.39%, respectively. The negative operating costs indicate that export revenue exceeds operating expenses. Notably, HRES‐4 outperforms HRES‐3, achieving the lowest LCOE due to the substantially lower capital cost of the second‐life battery while maintaining comparable operational performance. These findings demonstrate that repurposed EV batteries are not merely a lower‐cost substitute for new batteries but a key enabler of economically viable PV‐based hybrid energy systems, delivering comparable energy management capabilities with significantly improved lifecycle economics.

### 3.4 Environmental Impact

The environmental performance of the investigated configurations is evaluated in this section, and the results are summarized in Table 7. Significant differences are observed between the grid‐dependent systems (Grid, HRES‐1, and HRES‐2) and the renewable‐integrated configurations (G‐PV, HRES‐3, and HRES‐4) in terms of carbon emissions, emission mitigation, and lifecycle environmental impacts. The storage‐free G‐PV configuration serves as the reference case for quantifying the environmental contribution of the SLB. It should be noted that the reported emission metrics are based on different time scales. The produced emissions (PE), calculated using Equation (10), represent the cumulative CO_2_ emissions over the 25‐year project lifetime, whereas the mitigated emissions (PM), calculated using Equation (11), are expressed as annual CO_2_ reductions based on yearly PV electricity generation. Consequently, these quantities should not be directly compared or combined. For consistency, the lifetime PE values correspond to annualized emissions of approximately 818.3, 544.1, 942.1, 886.9, 384.2, and 331.2 tCO_2_/year for the Grid, G‐PV, HRES‐1, HRES‐2, HRES‐3, and HRES‐4 configurations, respectively, providing a consistent basis for comparison with the annual PM values.

**TABLE 7:** Environmental Impact for the different configurations.

Quantity	Grid	G‐PV	HRES‐1	HRES‐2	HRES‐3	HRES‐4
*CExC* (MWh)	8479.38	7538.28	12045.46	10455.06	4245.24	2722.33
Grid supply energy (MWh/25 yr)	15088.05	8133.25	15088.05	15088.05	6821.83	6815.83
PV output (MWh/yr)	0	602.09	0	0	1337.99	1337.99
RF (%)	0	63.2	0	0	80.9	80.9
$PE_{CO_{2}}$ (tons)	20456.53	13602.9	23551.88	22171.42	9606.2	8279.12
*PE_GWP_* (tons.CO_2_.eq. yr^−1^)	21493.5	14292.44	24745.76	23295.3	10093.2	8698.8
$PM_{CO_{2}}$ (tons/yr)	0	522.6	0	0	1161.38	1161.38
*PM_GWP_* (tons.CO_2_.eq. yr^−1^)	0	551.5	0	0	1225.6	1225.6
Carbon payback time (yr)	0	26.03	0	0	8.27	7.13
$CT_{CO_{2}}$($/yr)	409.13K	272.06K	471.04K	443.43K	192.12K	165.58K
$CRI_{CO_{2}}$($)	0	13.07	0	0	29.03k	29.03k

HRES‐1 was identified as the highest‐emitting system among all the configurations. It produces approximately 23,551.88 tons CO_2_/year and 24 745.76 tons CO_2_‐eq/year in terms of Global Warming Potential (GWP). These values are higher by 13.14%, 5.86%, 59.21%, and 64.85% compared to the Grid, HRES‐2, HRES‐3, and HRES‐4 systems, respectively. Consequently, this results in a carbon tax of approximately $471.04K. In contrast, HRES‐4 is ranked the most environmentally sustainable configuration due to its lower emissions and greater emission mitigation potential. The results indicate that this system mitigates approximately 1161.38 tons CO_2_/year and 1225.6 tons CO_2_‐eq/year in terms of CO_2_ and GWP, respectively, corresponding to a carbon credit of approximately $29.03K and a high renewable fraction of 80.9%. These mitigation values are comparable to those of HRES‐3, primarily because of similar PV energy production.

The carbon payback time (CPT) reported in Table 7 requires clarification, as it differs from the conventional definition. In this study, CPT was calculated as the ratio of project lifetime produced emissions (PE) to annual mitigated emissions (PM). Unlike the conventional carbon payback time, which compares the embodied carbon of system construction with the annual operational emissions avoided, the present metric represents the ratio of lifetime grid‐related emissions to annual emission mitigation and should be interpreted accordingly. The G‐PV reference case distinguishes the environmental contributions of PV generation from those of battery storage. Compared with the grid‐only configuration, PV integration alone reduced annual CO_2_ emissions from 20 456.53 to 13 602.9 tCO_2_/year (33.5%), mitigated 522.6 tCO_2_/year, and increased the renewable fraction to 63.2%. Integrating the second‐life battery (SLB) further decreases annual emissions to 8279.12 tCO_2_/year (an additional 39.1% reduction), increases the renewable fraction to 80.9%, and more than doubles the annual mitigated emissions to 1161.38 tCO_2_/year by enabling greater utilization of PV generation. Consequently, the CPT decreases from 26.03 years for G‐PV to 7.13 years for HRES‐4, while the CExC is reduced from 7538.28 to 2722.33 MWh. Compared with HRES‐3, HRES‐4 further reduces the CPT from 8.27 years to 7.13 years. Because both configurations employ identical PV capacities and achieve the same annual emission mitigation, this improvement arises solely from the lower emission term derived from the reduced acquisition cost of the second‐life battery through Equations (10) and (12), rather than from a directly quantified reduction in embodied carbon.

In terms of practical environmental equivalence, the results show that the mitigated carbon emissions corresponded to removing approximately 214.86 cars from the road, avoiding 548.43 L of gasoline consumption, reducing crude oil usage by 2671.17 barrels, recycling 400.68 tons of waste, and offsetting emissions equivalent to 255.5 acres of forest.

The environmental results demonstrate that second‐life batteries provide the greatest benefits when integrated with PV generation. The G‐PV configuration reduces grid electricity consumption by 46.10%, leading to corresponding reductions in CExC (11.10%), $PM_{CO_{2}}$, *PM_GWP_*, and carbon tax (33.50%). In contrast, HRES‐1 (new battery) and HRES‐2 (second‐life battery) increase environmental impacts because, without PV, the batteries only store grid electricity. However, HRES‐2 consistently performs better than HRES‐1, with smaller increases in CExC (23.30% vs. 42.06%) and emissions (8.38% vs. 15.13%). When combined with PV, HRES‐4 achieves the best environmental performance, reducing CExC by 67.89%, grid electricity consumption by 54.83%, and $PM_{CO_{2}}$, *PM_GWP_*, and carbon tax by 59.53%. Compared with HRES‐3, the second‐life battery delivers greater reductions in resource consumption and emissions while maintaining similar grid energy savings, highlighting its ability to enhance PV utilization with lower lifecycle impacts.

It should be emphasized that the environmental indicators reported in this study, including annual emission reduction, mitigated emissions, and the associated emission ratios, are operational rather than full life‐cycle indicators. They quantify the emissions avoided through reduced grid electricity consumption but do not explicitly account for the embodied environmental burdens associated with battery manufacturing, refurbishment, transportation, or end‐of‐life management. Consequently, two systems with similar operational emissions may differ substantially in their overall environmental impacts depending on the embodied impacts of their constituent components.

This limitation arises directly from the methodology presented in Section 2.4. The produced‐emission term in Equation (10) is derived from the cumulative exergy consumption (CExC), which is estimated from the total capital cost using Equation (12). By converting the life cycle expenditure into an equivalent quantity of grid electricity, the method implicitly assigns the same emission intensity per unit cost to all system components, irrespective of their actual embodied carbon. Consequently, the environmental advantage of HRES‐4 over HRES‐3 is represented primarily by the lower acquisition cost of the second‐life battery rather than by the explicit accounting of avoided battery manufacturing. Because both configurations employed the same PV capacity, they achieved identical annual avoided emissions (1161.38 tCO_2_/year), whereas the remaining environmental differences originated from the cost‐based CExC approach.

A comprehensive process‐based life cycle assessment would likely strengthen, rather than weaken, the environmental case for second‐life batteries. Under the commonly adopted cut‐off allocation approach, the manufacturing impacts of a retired EV battery are assigned to its first life, allowing the second‐life application to inherit little or no manufacturing burden from the environment. Because the present study did not explicitly model these life cycle stages, the reported environmental benefits should be interpreted as being conservative estimates. A detailed process‐based life cycle assessment remains an important direction for future work, which is discussed further in Section 3.8.

Overall, these results ranked HRES‐4 as the most sustainable. Benchmarking against the storage‐free G‐PV case confirms that while PV and electricity export account for a large share of the absolute emission reductions, the second‐life battery is responsible for the higher renewable fraction and for more than doubling the mitigated emissions. This ranking is established based on the operational indicators available here, and the distinct environmental case for second‐life deployment, which rests on avoided manufacturing rather than displaced grid electricity, remains outside the boundary of this assessment.

### 3.5 Sensitivity Analysis

**FIGURE 7:**
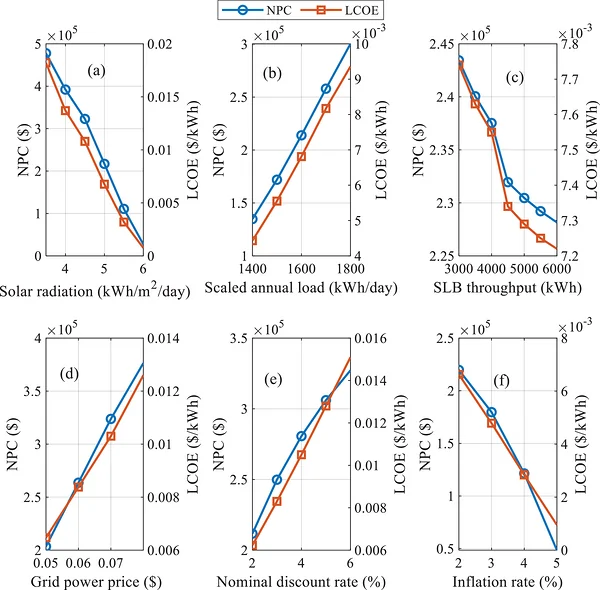
Effects of (a) solar radiation, (b) Load demand (c) SLB throughput, (d) Grid power price, (e) nominal discount rate, and (f) inflation rate on economic system performances.

The main objective of this study was to investigate the robustness of integrating SLBs into HRES under varying operating conditions and parameter uncertainties. To achieve this objective, a sensitivity analysis based on the optimization results was conducted, and the findings are presented in this section. Figure 7 illustrates the impact of variations in key operating and economic parameters on the NPC and LCOE. The results indicate that the economic performance of the proposed HRES‐4 configuration is highly sensitive to these parameters. In general, changes in the variables considered led to noticeable variations in both NPC and LCOE, with varying magnitudes of impact. For instance, an increase in solar radiation and inflation rate leads to a reduction in both NPC and LCOE. Specifically, increasing solar radiation from 3.5 to 6 kWh/m^2^/day reduces the NPC and LCOE by $450 487 and $0.017452/kWh, respectively (see Figure 7a). This improvement is attributed to the enhanced power output from the PV system, which reduces dependence on the grid and improves overall economic feasibility. Similarly, increasing the inflation rate from 2% to 5% decreases the NPC and LCOE by $171 201 and $0.00568/kWh, respectively (see Figure 7f), as future costs are discounted more heavily over the long term, reducing their present value.

In contrast, increases in load demand, grid electricity price, and nominal discount rate negatively affect the economic performance of HRES‐4, as shown in Figure 7b,d,e, respectively. As illustrated in Figure 7b, increasing the load demand from 1400 to 1800 kWh/day results in increases of $165 557 in NPC and $0.00493/kWh in LCOE. This is primarily due to the need for a larger system capacity and increased grid dependence, which raise both capital and operating costs. These findings highlight that the system cost is highly sensitive to demand growth, making load management a critical economic factor. Similarly, an increase in grid electricity price leads to a higher NPC and LCOE (see Figure 7d). This is because approximately 17% of the system's energy demand is still supplied by the grid, meaning that higher tariffs directly increase operating costs. However, the rate of increase remains moderate because of the contribution of PV generation, which limits exposure to grid price fluctuations. This indicates that the economic impact of grid price variations depends strongly on the share of grid energy in the system.

The nominal discount rate is another important parameter for assessing economic feasibility. As shown in Figure 7e, an increase in the discount rate results in a higher NPC and LCOE. Specifically, a 4% increase leads to increases of $115 490 in NPC and $0.00887/kWh in LCOE. This occurs because a higher discount rate lowers the present value of future cash flows while increasing the cost of capital, making long‐term investments such as PV and storage systems more expensive.

The SLB throughput was one of the key parameters investigated in this study, and its influence on the economic feasibility of the system was assessed, as shown in Figure 7c. The findings indicate that increasing the initial SLB throughput reduces both the NPC and LCOE. This trend reflects an improvement in the SoH of the batteries used, as a higher throughput implies a greater usable capacity and energy‐processing capability. Consequently, capital costs are distributed over a larger amount of delivered energy, thereby improving cost efficiency.

**FIGURE 8:**
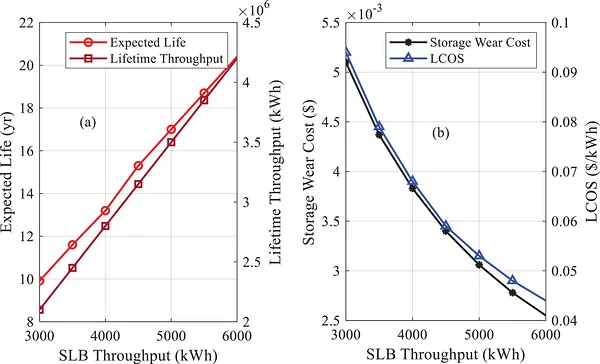
Influence of SLB Throughput on the Performance and Economic Metrics of the Storage System.

For instance, increasing the SLB throughput from 3000 to 6000 (corresponding to approximately 65%–130% of the baseline throughput of a new battery‐based containerized storage system) results in reductions of $15 289 in NPC and $0.00052/kWh in LCOE. However, these reductions remain relatively modest compared to other parameters, indicating that SLB throughput has a limited impact on improving the overall economic feasibility of the proposed HRES‐4 system. To further investigate the benefits of SLBs, a more detailed analysis of throughput variation was conducted. Its impact on the performance and economic metrics of the containerized storage system was evaluated, with the corresponding results presented in Figure 8.

Figure 8a illustrates the impact of SLB throughput on battery expected lifetime and cumulative lifetime throughput. The results show that both parameters increase almost linearly with SLB throughput. At the maximum SLB throughput of 6000 kWh, the expected lifetime and total lifetime throughput reached approximately 20.4 years and 4 200 000 kWh, respectively. This indicates that higher SLB throughput enhances the total energy delivered over the battery's lifetime, improving overall utilization despite degradation effects. In contrast, lower SLB throughput results in a shorter operational lifetime, suggesting that batteries selected for second‐life applications should have a relatively high SoH to ensure adequate performance and longevity.

Figure 8b illustrates the impact of the SLB throughput on storage wear cost and LCOS. The results indicate that increasing the SLB throughput enhances the economic feasibility of the storage system. Specifically, a higher throughput reduces both the storage wear cost and LCOS. As the SLB throughput increases from 3000 kWh to 6000 kWh, the storage wear cost and LCOS decrease by approximately $0.00255/kWh and $0.05/kWh, respectively. This improvement can be attributed to the fact that a higher throughput distributes fixed and degradation‐related costs over a larger amount of delivered energy, thereby reducing the cost per unit of energy.

### 3.6 Discussion

#### 3.6.1 Electrochemical Performance and Functional Viability of Second‐Life Batteries

The results confirm that SLBs can deliver performance comparable to that of newly installed batteries when deployed in PV‐coupled HRES configurations. This can be attributed to the stable cycling behavior of SLBs under moderate depth‐of‐discharge and low C‐rate conditions, which are typical of PV‐coupled storage systems, despite a reduced SoH of approximately 80%. The findings further indicate that the operational lifetime during the second‐life phase is broadly comparable to that of new batteries.

A key observation is that the second‐life operational phase exhibits a degradation trajectory that is less aggressive than that in electric vehicle applications. This is mainly due to the absence of high‐power transients and rapid cycling conditions typically encountered in mobility use. Although SLBs demonstrate slightly higher storage wear costs and reduced lifetime compared to new batteries, the controlled operating regime characterized by daytime charging and nighttime discharging creates a favorable cycling environment. This mitigates degradation mechanisms such as lithium plating and thermal instability, thereby supporting reliable long‐term operation.

#### 3.6.2 Throughput–Degradation Trade‐Off and Operational Optimization

The analysis highlights a critical relationship between energy throughput and battery degradation. Increased throughput improves system utilization and reduces the LCOS; however, it simultaneously accelerates capacity fade due to higher cycling intensity. This establishes a clear trade‐off between maximizing energy delivery and preserving battery longevity. However, in stationary applications, this trade‐off is moderated by relatively stable operating conditions. The absence of rapid charge–discharge cycles limits severe degradation, resulting in more predictable performance over time. The near‐linear relationship between throughput and lifetime energy delivery suggests that SLBs exhibit consistent degradation patterns under controlled conditions. This reinforces the importance of implementing health‐aware dispatch strategies that dynamically balance utilization with remaining battery capacity to optimize the overall system performance.

#### 3.6.3 Lifecycle Extension and Environmental Implications

From an environmental perspective, SLB integration provides a meaningful reduction in lifecycle emissions by extending battery service life and delaying recycling processes. Recycling lithium‐ion batteries remains energy‐intensive and associated with significant environmental impacts, particularly in pyrometallurgical and hydrometallurgical processes [52]. Rather than eliminating these impacts, SLBs redistribute them over an extended timeframe. This delay reduces the annualized environmental burden and improves overall lifecycle efficiency. In addition, extending battery use provides time for advancements in recycling technologies, potentially enabling more efficient, lower‐emission recovery processes in the future. However, it is important to recognize that the environmental benefits of SLBs depend heavily on the second‐life duration. If the operational lifespan is too short or if system inefficiencies increase energy losses, the expected environmental gains may be partially offset. Therefore, lifecycle optimization remains critical in evaluating the true sustainability of SLB systems.

#### 3.6.4 System Integration Challenges and Battery Heterogeneity

A major barrier to large‐scale SLB deployment is the inherent heterogeneity of battery conditions. Variations in state‐of‐health, internal resistance, and residual capacity introduce significant complexity in system integration and may compromise operational reliability if not properly managed [53]. To overcome these challenges, robust system‐level strategies are required, including health‐based sorting and classification of battery modules, grouping cells into homogeneous clusters, and implementing advanced BMS with real‐time monitoring capabilities. In addition, dynamic balancing and SoH‐aware control strategies are essential to ensure stable and efficient operation across diverse battery conditions. Thermal management also emerges as a critical design consideration, as aged batteries tend to exhibit nonuniform heat generation, which can accelerate degradation and increase safety risks. In this context, the containerized architecture proposed in this study helps mitigate these challenges by facilitating modular system integration, effective thermal management, and systematic battery control. However, the SoH assumption adopted in Section 2.2 does not explicitly capture the heterogeneity of retired battery cells. Consequently, the reported system‐level results assume that appropriate cell sorting, clustering, and SoH‐aware balancing strategies are successfully implemented. The findings therefore represent the performance potential of a well‐managed containerized second‐life battery system rather than that of an unsorted collection of retired cells. This modelling assumption constitutes a limitation of the present study and is discussed further in Section 3.7. However, further advancements in integrated BMS technologies and thermal optimization approaches remain necessary to ensure safe, reliable, and long‐term operation of SLB‐based energy storage systems.

#### 3.6.5 Economic Trade‐Offs and Application Suitability

The economic advantage of SLBs is primarily driven by their significantly lower capital cost, typically around 50% of that of new batteries. This substantial reduction in upfront investment plays a critical role in enhancing overall system economics, particularly in PV‐integrated configurations where storage is coupled with renewable generation. The results indicate that HRES‐4 achieved the lowest NPC and LCOE among all configurations, confirming that the cost savings associated with SLBs can offset their shorter operational lifetime and relatively higher degradation rates under the studied conditions. However, this economic benefit is strongly dependent on the application context. SLBs are most viable in systems characterized by low‐to‐moderate cycling intensity, stable and predictable load profiles, and limited peak power demand. Under these conditions, degradation remains manageable, allowing the system to fully capitalize on the reduced capital expenditure. In contrast, high‐power or highly dynamic applications tend to accelerate battery aging, which can diminish the economic advantage of SLBs over time. These findings suggest that SLBs should not be viewed as a universal substitute for new batteries, but rather as a context‐specific solution whose viability depends on aligning their technical characteristics with appropriate operational environments.

#### 3.6.6 Circular Economy Potential and Energy System Transformation

The deployment of SLBs supports the transition toward a circular energy economy by enabling the reuse of valuable battery materials and reducing waste. This approach creates opportunities for new industrial value chains focused on battery collection, diagnostics, repurposing, and redeployment. In addition, SLBs introduce a feedback mechanism into the electric vehicle ecosystem. The residual value of used batteries can offset the initial cost of EV ownership, indirectly supporting the expansion of electric mobility in the country. This interconnected value chain highlights the strategic role of SLBs beyond energy storage, positioning them as key enablers of sustainable energy transitions.

#### 3.6.7 Policy Implications and Global Relevance

Beyond the system‐level results, these findings have broader implications for the global energy transition and circular economy. With retired electric‐vehicle batteries projected to reach 314 GWh by 2030 [54], second‐life deployment offers a scalable response to a fast‐growing waste stream while simultaneously lowering the cost barrier to renewable storage. The roughly 50% capital‐cost reduction relative to new batteries, combined with the operational emission reductions reported in Section 3.4, demonstrates that repurposing can deliver both economic and operational environmental value rather than forcing a trade‐off between them. It should be noted that this environmental claim rests on operational emissions only; the embodied burden of manufacturing, refurbishment, transport, and end‐of‐life treatment is outside the boundary adopted here (Sections 2.4 and 3.8). For policymakers, this strengthens the case for circular‐economy frameworks that formalize second‐life batteries through state‐of‐health certification standards, traceability and labeling requirements, and clear end‐of‐second‐life recycling obligations, so that residual battery value is captured safely and reliably at scale. The relevance is particularly acute for tropical and emerging grids: the high and stable solar resource modeled in Kuala Lumpur enabled second‐life storage to cut grid dependency by more than 54% and turn a residential community into a net energy exporter, indicating that comparable solar‐rich regions in the Global South could adopt this pathway to expand affordable, low‐carbon storage without the upfront cost of new battery systems. Realizing this potential at scale will, however, depend on parallel progress in standardized sorting protocols, advanced battery management for heterogeneous cells, and lifecycle‐integrated recycling strategies, underscoring that the technical, economic, and policy dimensions of second‐life deployment must advance together.

### 3.7 Comparison with Recent Representative Studies

Techno‐economic assessments of second‐life batteries using HOMER have been reported widely. Therefore, Table 8 compares the present study with four recent representative studies to highlight key distinctions in terms of methodology, application, and regional context.

**TABLE 8:** Comparison with recent HOMER‐based second‐life battery studies.

Study	Tool/case	System and storage	Storage‐free PV reference	Attribution of benefit	Key reported outcome
Sarker et al. [55]	HOMER Pro + Simulink, Cyberjaya, Malaysia.	15 kW PV; 50 kWh SLB; EV charging station.	No	Not separated; benefits ascribed to SLB.	LCOS at $0.08/kWh, 40% below the new‐battery benchmark; 78% self‐sufficiency; measured SoH, 75%, 3%–4% loss over 100 cycles.
Katche et al. [56]	HOMER Pro, Moi University, Kenya.	PV + grid, lead‐acid battery, and institutional building.	Yes	PV was isolated, and storage was evaluated against it.	Grid/PV without storage optimal (LCOE 8.78 vs. 10.49 KSH/kWh); adding a new battery worsened both LCOE and NPC.
Ozcan et al. [57]	HOMER Grid + BEopt: Antalya and Istanbul, Türkiye.	5.9‐7.5 kW PV; 9–11.5 kWh SLB; net‐zero houses	Yes	Ranking of battery types reported.	Ranking: PV‐SLB > PV‐no battery = PV‐new Li‐ion > PV‐new lead acid; SLB raises NPV by 15%–21%.
Bhatt et al. [58]	HOMER Pro; AIT Campus, Thailand.	Four hybrid models, fresh and second‐life batteries; campus microgrid	No	Not separated	Optimal net‐zero microgrid configuration identified; SLB sizing sensitivity is reported.
This work	HOMER Pro, residential community, Kuala Lumpur, Malaysia.	1,000 kW PV; 896 kWh containerized SLB (538 kWh usable); grid connection.	Yes (G‐PV)	Quantified: PV alone vs. incremental storage.	PV alone gives RF 63.2%; SLB raises RF to 80.9% by removing the absorption limit; HRES‐4 NPC 39% below G‐PV, while the new‐battery case is level with it.

The closest comparator is the study by Sarker et al. [55], which assessed a second‐life battery–PV EV charging station in Malaysia using HOMER Pro and MATLAB Simulink, reporting a storage cost approximately 40% lower than that of a new battery. Unlike the present study, it included experimental battery characterization and a Monte Carlo environmental analysis. However, because PV and storage were introduced simultaneously, the individual contribution of the second‐life battery could not be isolated.

This limitation was addressed in the present study by introducing a storage‐free G‐PV reference case. Similar comparisons by Katche et al. [56] and Ozcan et al. [57] showed that grid‐connected PV systems without storage can outperform systems with new batteries, indicating that battery storage is not always economically justified in the long run. The present results confirm this finding: the G‐PV configuration achieves a 63.2% renewable fraction and reduces the 25‐year cumulative cost by 47% relative to the grid‐only system, while HRES‐3 (PV + new battery) provides little additional economic benefit.

The key contribution of this study is demonstrating when second‐life batteries become valuable. Comparing HRES‐4 with G‐PV shows that low‐cost second‐life storage enables higher PV penetration by absorbing surplus generation, increasing the renewable fraction from 63.2% to 80.9%, and transforming the system into a net electricity exporter. This benefit is not achieved with new batteries because of their higher capital cost.

### 3.8 Limitations and Future Research Directions

The most immediate limitation of this study is the uniform SoH representation of the second‐life battery pack, as described in Section 2.2. The simulations assume a uniform initial SoH of 80%, identical degradation behavior, and identical electrical characteristics for all cells, resulting in deterministic point estimates without quantified uncertainty. Addressing this limitation requires a stochastic, distribution‐based framework in which residual capacity and internal resistance are sampled from empirical distributions of retired EV cells and propagated through a string‐level electrical model. Such an approach would quantify the effects of cell‐to‐cell variability on usable capacity, round‐trip efficiency, service life, and the resulting NPC, LCOE, and LCOS, while also assessing the benefits of health‐based cell sorting.

This analysis was not performed for two reasons. First, HOMER Pro represents the battery as a single equivalent storage unit and cannot model cell‐level variability or stochastic pack behavior. Second, no measured SoH distribution was available for the retired cells considered in this study, and assuming one would introduce unverified uncertainty into the results. Consequently, the proposed stochastic analysis depends on the empirical characterization of representative retired‐cell populations, such as that reported by Sarker et al. [55], and is identified as a key direction for future research. Future work should also incorporate physics‐based degradation models and real‐time SoH estimation integrated with adaptive energy management strategies.

The second limitation concerns environmental assessment. As described in Section 2.4, the analysis considered only operational emissions, with produced emissions derived from the system cost rather than a process‐based life‐cycle inventory. Consequently, the methodology cannot distinguish the contributions of manufacturing, refurbishment, transportation, or end‐of‐life treatment, and the environmental advantage of second‐life batteries is largely driven by their lower acquisition costs. A cradle‐to‐grave life‐cycle assessment using a detailed emission factor inventory would explicitly account for the avoided manufacturing burden of repurposed batteries and is therefore expected to strengthen the environmental case for second‐life applications. Future research should also establish standardized testing and certification frameworks and integrate recycling pathways into life cycle optimization models to improve the reliability, sustainability, and commercial viability of second‐life battery energy storage systems.

## 4 Conclusion

This study presents a comprehensive techno‐economic and environmental assessment of integrating containerized SLBs into grid‐connected HRES. A HOMER Pro‐based optimization framework was developed to evaluate multiple system configurations under realistic operating conditions, enabling a comparative analysis between new and second‐life battery deployments.

The results demonstrate that the PV‐integrated SLB configuration (HRES‐4) consistently outperforms alternative configurations across key performance indicators. Specifically, HRES‐4 achieved the lowest NPC and LCOE, at approximately $239.6K and $0.00762/kWh, respectively, reflecting the significant capital cost advantage associated with second‐life batteries. Despite exhibiting slightly higher degradation rates and a reduced operational lifetime compared to new batteries, SLBs maintain comparable energy throughput and cycling performance, particularly under moderate residential load profiles. This can be attributed to the high utilization of the storage system in HRES‐4, exceeding 207 MWh per year, and its ability to surpass 230 equivalent full cycles annually, thereby confirming its technical viability. From an energy system perspective, the integration of PV and SLBs reduces grid dependency by more than 54% and enables substantial energy exports (>820 MWh/year), effectively transforming the system into a net energy producer. The results further indicate that storage‐only configurations without renewable integration are economically unfavorable, as they increase capital expenditure without delivering proportional operational benefits. Environmentally, the use of SLBs contributes to significant CO_2_ emission reductions and supports circular economy principles by extending battery lifecycle and delaying resource‐intensive recycling processes. The carbon payback period of approximately 7.13 years underscores the environmental feasibility of second‐life deployment under the conditions studied. Sensitivity analysis confirms that solar irradiance and load demand are the dominant factors influencing system performance, emphasizing the importance of site‐specific design and resource assessment. The findings validate that containerized SLB systems represent a cost‐effective and sustainable solution for enhancing RE integration in grid‐connected applications. However, their large‐scale deployment remains contingent on addressing key challenges, including state‐of‐health variability, standardized sorting protocols, and advanced battery management strategies for heterogeneous cells.

## Author Contributions

**Abdelhak Lekbir and Bessam Deboucha**: conceptualization, data curation, methodology, investigation, visualization, formal analysis, software, writing – original draft. **Abdullahi Mohamed Samatar**: conceptualization, investigation, formal analysis, writing – original draft. **Saad Mekhilef**: funding acquisition, validation, resources, project administration, writing – review and editing. **Kok Soon Tey**: validation, writing – review and editing. **Mehdi Seyedmahmoudian**: writing – review and editing. **Khalid Alqunun**: writing – review and editing.

## Conflicts of Interest

The authors declare no conflicts of interest.

## Data Availability

Data will be made available from the corresponding author upon reasonable request.
